# Identification of a novel cell cycle-related risk signature predicting prognosis in patients with pancreatic adenocarcinoma

**DOI:** 10.1097/MD.0000000000029683

**Published:** 2022-11-18

**Authors:** Dapeng Xu, Rong Qin, Ming Li, Jun Shen, Yongmin Mao, Kai Tang, Aiguo Zhang, Dafeng Wang, Yingzuo Shi

**Affiliations:** Department of Pediatric Surgery, The Affiliated Wuxi Children’s Hospital of Nanjing Medical University, Wuxi, China.

**Keywords:** cell cycle, LASSO, pancreatic adenocarcinoma, prognostic signature, TMB

## Abstract

Growing evidence have indicated that cell cycle-related genes (CRGs) play an essential role in the progression of pancreatic adenocarcinoma (PAAD). Nevertheless, the application of CRGs in estimating the prognosis of PAAD patients is still lacking. This study aimed to establish a risk signature based on CRGs that can predict patients’ overall survival for PAAD.

**Methods:**

The expression and corresponding clinical data of PAAD patients from The Cancer Genome Atlas database and 200 cell cycle-related genes from the MSigDB were used for the generation and validation of the signature. LASSO Cox regression was applied to build the prediction model. The diagnostic value of signature was evaluated by receiver operating characteristic curves. Univariate and multivariate regression was used to construct the nomogram providing the clinicians a useful tool.

**Results:**

A total of 103 CRGs were identified. Seven genes (RBM14, SMAD3, CENPA, KIF23, NUSAP1, INCENP, SMC4) with non-zero coefficients in LASSO analysis were used to construct the prognostic signature. The 7-gene signature significantly stratified patients into high- and low-risk groups in terms of overall survival, and the area under the receiver operating characteristic curve of 5-year survival reached 0.749. Multivariate analysis showed that the signature is an independent prognostic factor. We then mapped a nomogram to predict 1-, 3-, and 5-year survival for PAAD patients. The calibration curves indicated that the model was reliable. Finally, we discovered that TP53 and KRAS mutated most frequently in low and high-risk groups, respectively.

**Conclusion:**

Our findings suggested that the seven genes identified in this study are valuable prognostic predictors for patients with PAAD. These findings provided us with a novel insight that it is useful for understanding cell cycle mechanisms and for identifying patients with PAAD with poor prognosis.

## 1. Introduction

Pancreatic adenocarcinoma (PAAD), which mortality closely parallels incidence, is lethal and aggressive with a 5-year survival rate of only about 6% (ranging from 2% to 9%).^[1]^ Most patients with PAAD remain asymptomatic until the disease reaches an advanced stage. Surgical resection is the most effective therapy and it significantly increases the 5-year survival rate to 20%–30%. However, only <20% of patients have the opportunity for resection treatment as most patients are diagnosed at an advanced stage when there is metastasis.^[2]^ Despite improvements in surgery, radiotherapy, and chemotherapy, the survival rates for PAAD are still poor, even those with the same clinicopathological characteristics have different prognoses and treatment responses, and there are still lacking early diagnostic methods due to nonspecific symptoms and lacking effective testing identification.^[3]^ Hence, the development of a new strategy to reduce mortality and identify prognostic biomarkers during the early stage is an urgent task. Tang et al constructed a risk signature based on the ferroptosis-related genes and proved that immuno- and chemotherapy combined with a ferroptosis inducer is a feasible therapeutic approach for PAAD.^[4]^

An increasing amount of evidence demonstrates that the discovery and application of molecular biomarkers will improve the prognostic evaluation and identification of potential high-risk patients with PAAD, and it could also help provide insights into tumor progression and uncover potential new therapeutic targets. Multigene risk signatures derived from primary tumor biopsy can guide clinicians in designing an appropriate course of treatment. One study revealed a seven-gene prognostic model for PAAD based on transcriptome dysregulation, and this risk signature is stable in internal and external validation.^[5]^ However, there is still a deficiency in the performance of the newly developed biomarkers in PAAD. Therefore, finding a more efficient and sensitive signature is still a pressing problem to be solved.

The cell cycle is significantly associated with the growth and proliferation of cancer cells. Growing evidence have indicated that genes can change the process of tumors by regulating the cell cycle, thereby achieving the goal of targeted therapy. The cell cycle features have been used in endometrial cancer and gastric cancer.^[6,7]^ Both the two risk signatures have a high prognostic accuracy in predicting overall survival and distinguishing patients from those with the same characteristics.

In this study, we speculated that cell cycle-related genes and tumor mutation could provide prognostic value for patients with PAAD. A prognostic multigene signature with cell cycle-related genes was constructed in the TCGA cohort and compared the tumor mutation burden (TMB) in two different risk groups. We aimed to provide novel biomarkers that would be effective in predicting the prognosis and monitoring the tumor microenvironment in PAAD patients.

## 2. Materials and Methods

### 2.1. Data acquisition

RNA-Seq data and clinical information from pancreatic adenocarcinoma patients were downloaded from TCGA via the UCSC Xena platform (https://xena.ucsc.edu), which is routinely updated and integrated.^[8]^ The transcriptome profiling of RNA expression was obtained by RNA-seq and measured by fragments per kilobase of exon model per million mapped reads or FPKM values. The log2-based transformation was used for the normalization of RNA expression profiles. The cell cycle-related gene set was retrieved from “G2M checkpoint” from the GSEA database (http://www.gsea-msigdb.org/gsea/index.jsp). The mRNA expression of cell cycle genes in the TCGA database was extracted. Our study didn’t involve human beings or animals, so the approval of the Ethics Committee is not necessary for our study.

### 2.2. Identification of prognosis-associated differentially expressed CRGs

With the cut-off criteria set as |logFC| > 1 and *P* value < .05, we screened the DEGs via the “limma” R package.^[9]^ Then, a univariate Cox regression analysis was performed to identify prognosis-associated DE-CRGs. Hazard ratio (HR)<1 indicates better overall survival (OS) outcomes while HR>1 indicates worse OS outcomes. Genes with *P* < .05 were regarded as prognosis-associated cell cycle genes.

### 2.3. Functional enrichment analysis of the prognosis-associated DE-CRGs

Gene ontology (GO)^[10]^ and Kyoto Encyclopedia of Genes and Genomes (KEGG)^[11]^ pathway enrichment analyses were performed to explore the biological functions of the prognosis-related genes via the “clusterProfiler” R package. Adjusted *P* value < .05 was set as the significance threshold, and the enrichment analysis result maps were presented by the “ggplot2” and “GOplot” R packages.

### 2.4. Construction of the gene-related prognostic model

The LASSO regression model^[12]^ was used to identify the most accurate predictive genes. For example, if there were two different genes in parallel, LASSO would automatically filter out the secondary related one and assign the selected genes a value, which equals the regression coefficient in the classifier formulas. The risk score for the signature as computed using the formula:


$$\begin{array}{l} Risk\;score\;=\;\sum_{i=1}^{N}\left(Expi*Coei\right) \end{array}$$

where n represents the number of modules RNAs; Coef (i) is the coefficient; X(i) denotes the z-score-transformed relative N, Expi, and Coei represented the number of signature genes, gene expression level, and coefficient value, respectively. All patients in the cohort were classified into low- and high-risk groups based on the median of risk scores. Based on the median risk score, we divided the patients into high- and low-risk subgroups. In the two subgroups, each patient’s survival status, OS time, and gene expression profile were presented via the “pheatmap” and “survival” R packages. In addition, the Kaplan–Meier curve analysis was performed, and ROC curves were drawn to estimate the sensitivity and specificity of the prognostic signature.

### 2.5. Gene set enrichment analysis

We performed GSEA (http://www.broadinstitute.org/gsea/index.jsp) to determine if the identified gene sets were significantly different between cancer and normal groups. Next, we analyzed the expression levels of 20,530 mRNAs in PAAD samples and in adjacent noncancerous tissues. Finally, we determined functions for subsequent analysis by using normalized *P* values < .05.

### 2.6. Evaluation of clinical independence and construction of the nomogram

Next, we removed PAAD patients who lacked detailed clinicopathological information including survival status and time, age, grade, clinical stage, tumor grade, distant metastasis cancer status, and lymph node status. The clinicopathological characteristics and the CRGs expression data of the remaining patients were compared between the high- and low-risk subgroups and comprehensively displayed in the heatmap. Moreover, the clinical indexes and risk scores were included in univariate and multivariate Cox regression analyses to validate the independence of the risk model. ROC curves for the signature and other clinical features were used to assess the predictive efficacy of the model. In addition, the correlation between the CRGs from the risk model and the clinical index was also measured. Finally, we utilized the “rms” R package to consolidate the risk score and clinical characteristics for nomogram construction.

### 2.7. Clinical correlation analysis

Univariate regression analysis and multivariate regression analysis were used to identify factors (including gender, age, TNM stage, and risk score) affecting survival and independent prognostic factors in patients with PAAD. The correlation between survival-associated IRGs and clinicopathological characteristics was analyzed in R platform. *P* < .05 was considered to have a significant correlation.

### 2.8. Tumor mutation burden analysis

To explore the mutation landscapes of pancreatic adenocarcinoma, the somatic mutation data were processed and analyzed by R software (version 4.0.2) with the “maftools” package. TMB was defined as the total number of somatic mutations including somatic mutations, insertion-deletion mutations, coding, and base replacement of per million bases. The pancreatic cancer patients were separated into the low-TMB and high-TMB groups using the median value of TMB. To analyze the correlations between TMB and clinicopathological factors of patients with pancreatic cancer, we merged the TMB data with corresponding clinical information. The Wilcoxon rank-sum test was utilized for comparisons between two groups of clinical variables.

## 3. Results

### 3.1. Identification and functional analyses of survival-associated genes

**Figure 1.**
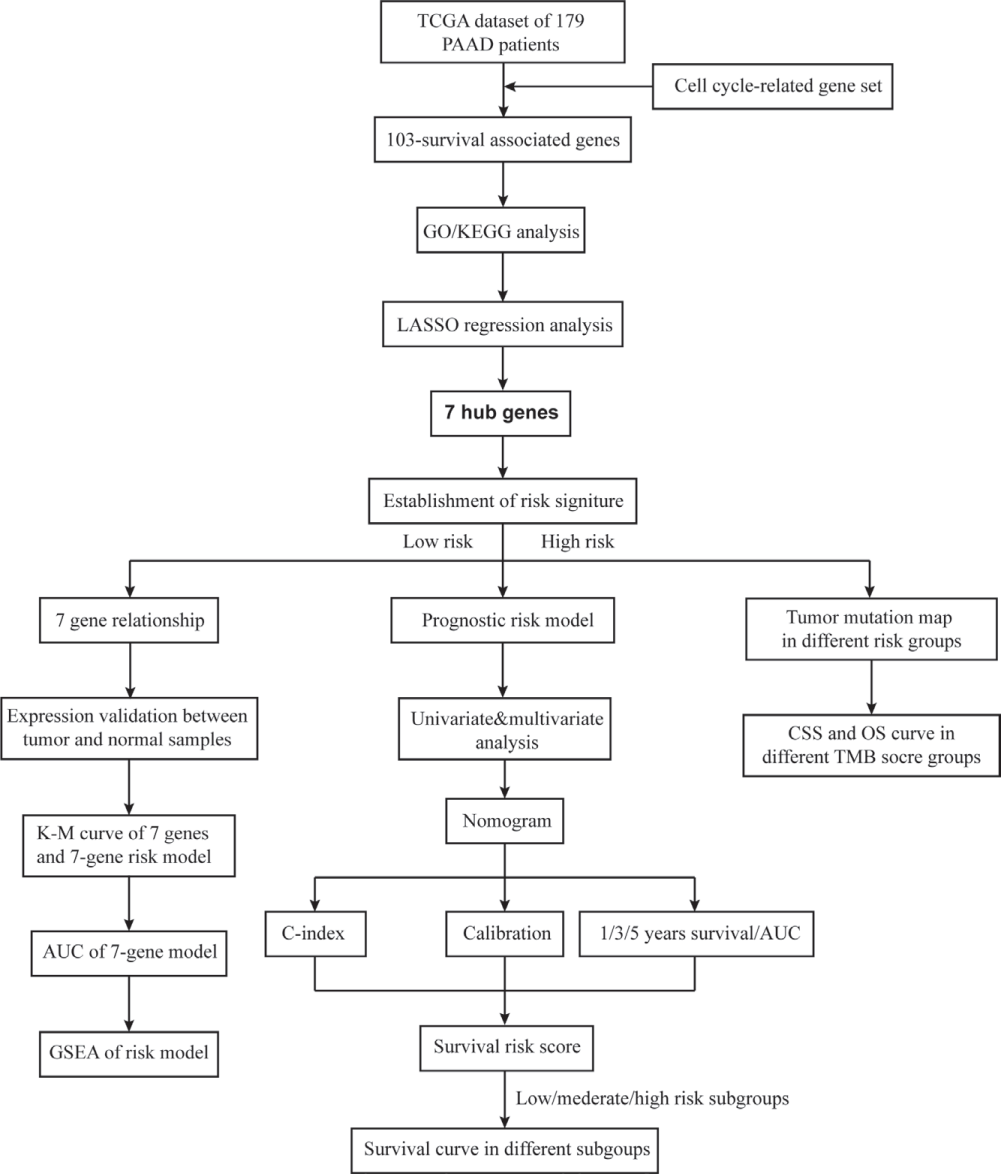
The flowchart of the study design. AUC = area under the ROC curve, DSS = disease-specific survival, GO = gene oncology, GSEA = gene set enrichment analysis, KEGG = Kyoto Encyclopedia of Genes and Genomes, K-M = Kaplan-Meier survival curve, LASSO = Least Absolute Shrinkage and Selection Operator, OS = overall survival, PAAD = pancreatic adenocarcinoma.

**Figure 2.**
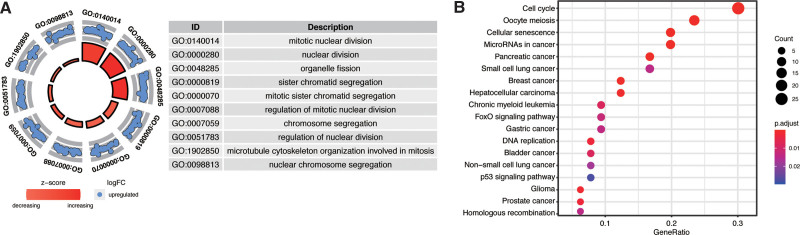
Gene annotation of 70 survival associated genes. (A) GO analysis. (B) The significantly enriched pathways of the genes determined by KEGG analysis. GO = gene ontology, KEGG = Kyoto Encyclopedia of Genes and Genomes.

The flow diagram for the present study was exhibited in Figure 1. Based on the gene set list from “Hallmark G2M checkpoint”, a total of 200 genes were extracted from the gene set. We then conducted the univariate Cox analysis to identify the CRGs that were associated with the survival and prognosis of patients because these genes may be the key biomarkers for evaluating patients. During further screening, we obtained 103 survival-associated CRGs for adenocarcinoma cancer (see Table S1, Supplemental Digital Content, http://links.lww.com/MD/G913, which illustrates the 103 survival-associated CRGs). To elucidate the potential function of these genes, GO and KEGG analyses were carried out. During enrichment analyses for these prognosis-associated CRGs, they were mainly found to be involved in “mitotic nuclear division” “nuclear division” and “organelle fissio” for GO enrichment (Fig. 2A), and “cell cycle” “FoxO signaling pathway” and “p53 signaling pathway” as shown in Kyoto Encyclopedia of Genes and Genomes (KEGG) (Fig. 2B). The above findings suggested that these CRGs might enrich these functions and play their roles through the pathways.

### 3.2. Construction of the risk assessment model

**Figure 3.**
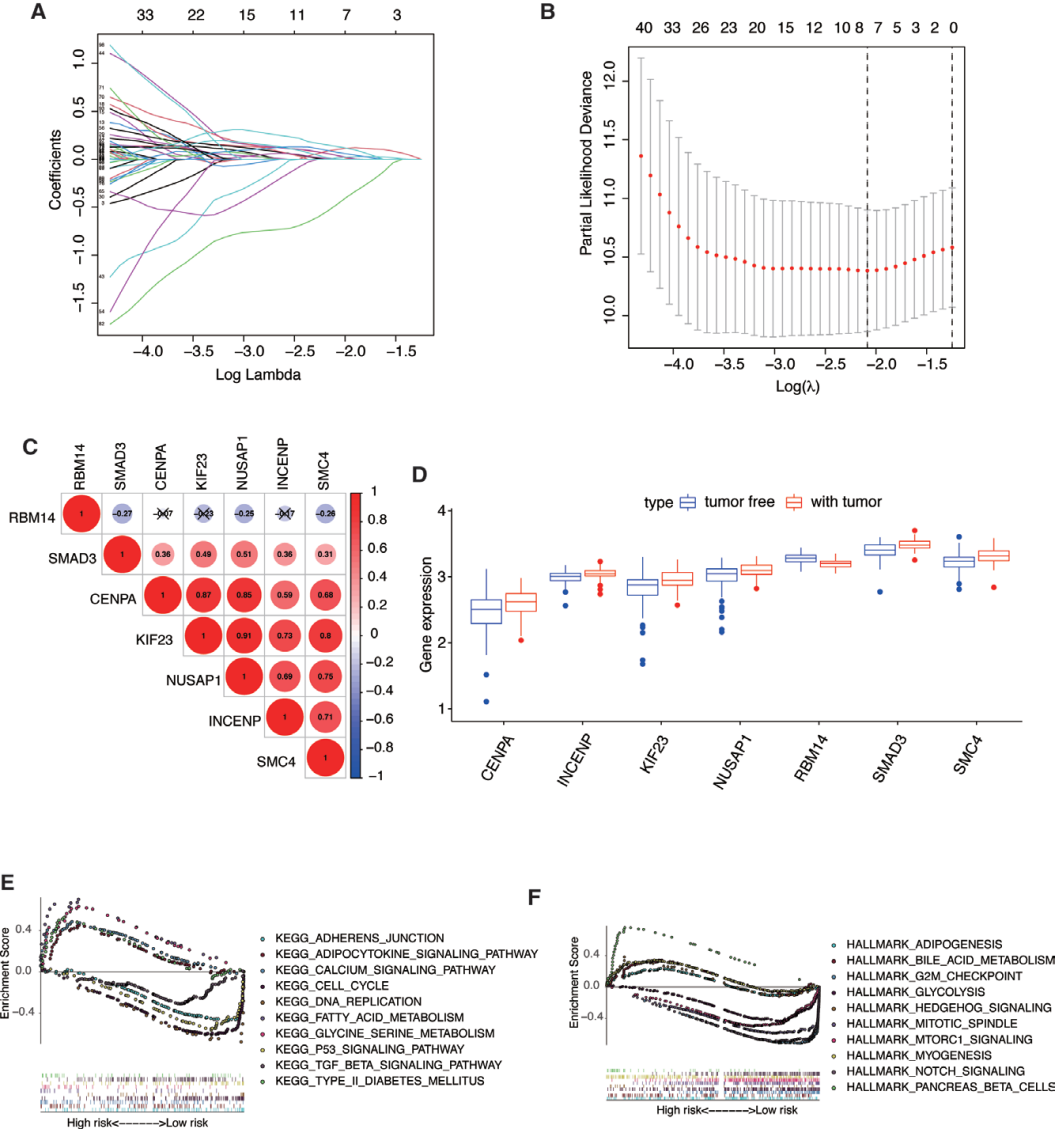
Ten-fold cross-validation for tuning parameter selection and a gene expression. (A) Plots of the ten-fold cross-validation error rates. (B) LASSO coefficient profiles of the seven cell cycle-related genes. (C) Relationships between the seven genes. (D) Gene expression of the seven genes in different tumor status. (E,F) GSEA analysis in low- and high-risk groups.

The results of the univariate Cox regression analysis of 103 genes were used in the LASSO regression to identify robust markers. A set of seven genes (RBM14, SMAD3, CENPA, KIF23, NUSAP1, INCENP, SMC4) and their coefficients were computed (Fig. 3A,B). Then, multivariate Cox regression analyses were performed, and a seven-gene model was constructed according to their coefficients (Table 1). Risk score=(CENPA*0.03992)+ (INCENP*0.04016) + (KIF23*0.10093) + (NUSAP1*0.01307)- (RBM14*0.4748) + (SMAD3*0.07337)+ (SMC4*0.13318). We further analyzed the relationship between the 7 genes (Fig. 3C). We found that they were significantly relevant, especially between KIF23 and NUSAP1, CENPA and KIF23, CENPA and NUSAP1, KAF23 and SMC4. The mRNA expressions of these 7 signature genes in different tumor statuses were compared using 179 samples (69 patients with tumor-free status and 110 with tumor status). The mRNA expression of SMAD3, CENPA, KIF23, NUSAP1, INCENP, and SMC4 were significantly up-regulated in patients with tumors (all *P* < .05). On the other hand, RBM14 was overexpressed in patients with tumor-free status (*P* < .05, Figure 3D). These 7 genes were also differentially expressed in different grade groups (see Figure S1, Supplemental Digital Content, http://links.lww.com/MD/G912, which showed the expression of seven genes in different grades). These findings suggested that these 7 signature genes may be involved in the development of PAAD. GSEA was then conducted to explore the biological functions enriched in high and low-risk groups. The results indicated that “adherens junction”, “adipocytokine signaling pathway”, “cell cycle”, “G2M checking”, and “glycolysis” were enriched in high-risk group (Fig. 3E). Meanwhile, “fatty acid metabolism”, “P53 signaling pathway”, “TGF beta signaling pathway”, “mitotic spindle”, and “Notch signaling pathway” were enriched in low risk group (Fig. 3F).

**Table 1:** Seven cell cycle associated genes and corresponding coefficient values.

Metabolic associated gene	Coefficient
CENPA	0.03992
INCENP	0.04016
KIF23	0.10093
NUSAP1	0.01307
RBM14	−0.47484
SMAD3	0.07337
SMC4	0.13318
Risk score	Low: <0.344
	High: ≥0.344

### 3.3. Kaplan–Meier survival analysis of 7 gene

**Figure 4.**
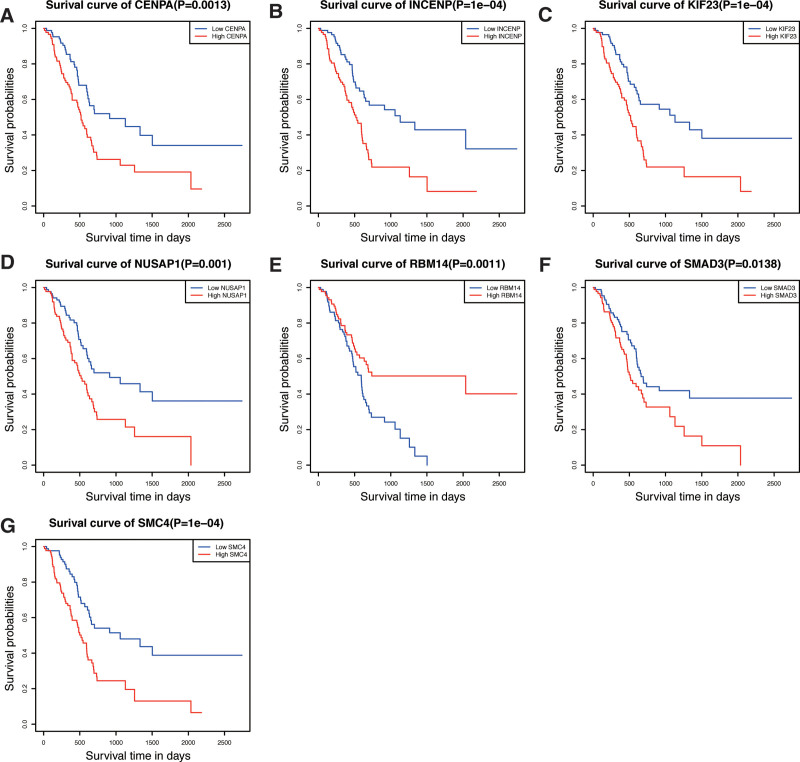
Prognostic significance of low and high expression of each of the 7 genes. (A) CENPA. (B) INCENP. (C) KIF23. (D) NUSAP1. (E) RBM14. (F) SMAD3. (G) SMC4.

We further tested the survival assessment model by Kaplan–Meier analysis in different subgroups of the 7 genes. Of the 7 subgroups classified by their expression, patients with high expression of SMAD3, CENPA, KIF23, NUSAP1, INCENP, and SMC4 had a worse prognosis compared with low expression (Fig. 4A–F). In contrast, the high expression group of RBM14 had a better prognosis than the low expression group (Fig. 4G). Therefore, each of the seven genes has certain reliability and practicability in evaluating the prognosis for PAAD patients.

### 3.4. Prognostic risk score indicated strong associations with clinical characteristics in PAAD

**Figure 5.**
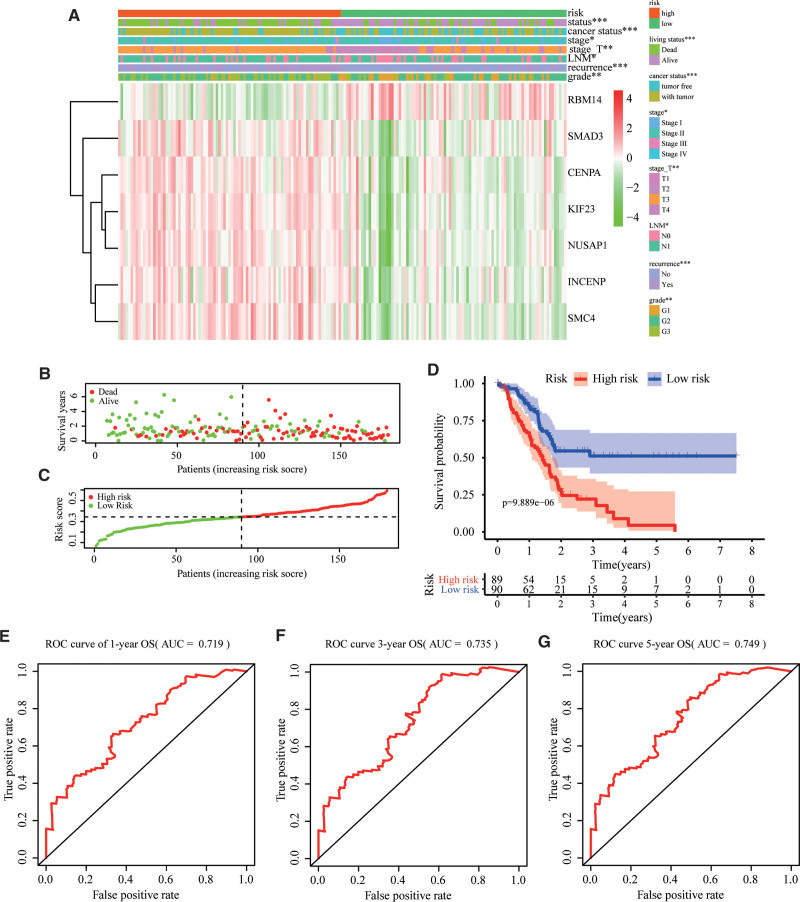
Correlation between the risk score and clinicopathological features. (A) Relationship between the risk model and clinical significance. (*P* value*** <.001, *P* value ** <.01, and *P* value * <.05). (B,C) Distribution of risk score and patient survival status of pancreatic cancer. (D) The Kaplan–Meier curve demonstrates that patients in the high-risk group have a poorer prognosis. (E–G) Time-dependent ROC curve of 1-, 3-, and 5-year analysis for survival prediction by the risk score.

The expression levels of the 7 genes and clinicopathological characteristics in high-risk and low-risk groups were presented in the heatmap (Fig. 5). The distribution of the seven genes across all samples showed that the patients in the high-risk group were likely to have a higher expression of SMAD3, CENPA, KIF23, NUSAP1, INCENP, and SMC4. In contrast, the patients in the low-risk group were inclined to have higher expression of RBM14. The results also showed that there were significant differences between the high-risk and low- risk groups in term of living status (*P* < .001), cancer status (*P* < .001), stage (*P* < .05), stage T (*P* < .01), LNM (*P* < .05), recurrence (*P* < .001), and grade (*P* < .01). The risk score of each PAAD patient was computed, and the patients were assigned to the low-risk (n=80) or high-risk (n=79) group based on the median cut-off value. Intuitively, the number of deaths was significantly higher in the high-risk group (Fig. 5B,C). The Kaplan–Meier analysis of all patients indicated that the survival of the patients in the low-risk group was significantly better than that of the patients in the high-risk group (*P* = 9.889e−06, Fig. 5D). The AUC of the survival assessment model of the 7-gene risk model was 0.719, 0.735, and 0.749 at 1-, 3-, and 5 years of OS (Fig. 5E–G).

### 3.5. The construction and validation of nomogram

**Figure 6.**
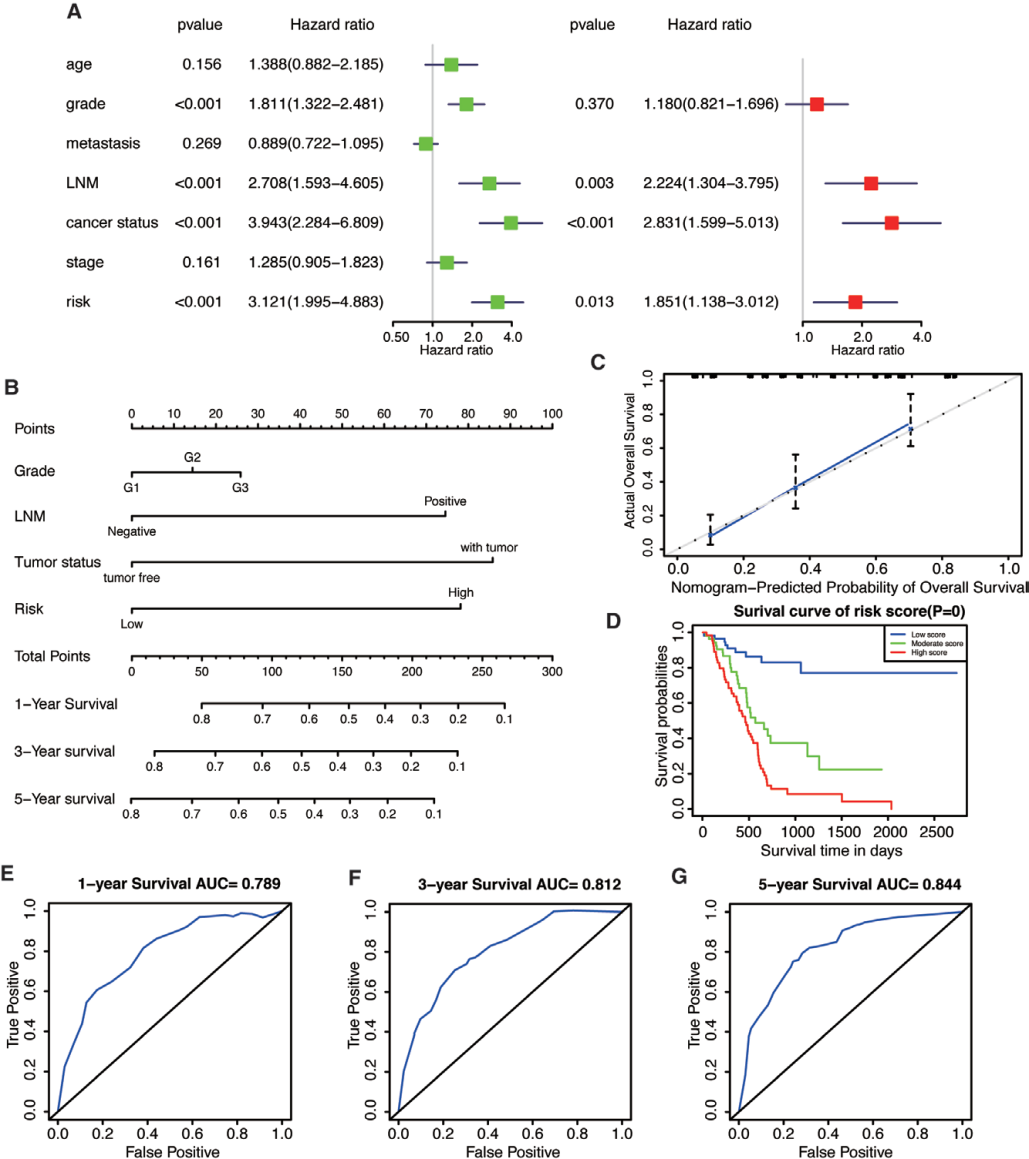
Nomogram to predict the probability of patients with PAAD. (A) Univariate and multivariate regression analyses of the prognostic value of clinicopathological features. (B) The nomogram to predict 1-, 3-, or 5-year OS in the PAAD patients. (C) The calibration plots for predicting patient 1-, 3-, or 5-year OS. (D) The Kaplan–Meier curves represent the survival probability of low, moderate, and high score group patients based on the nomogram. (E-G) The ROC of 1-, 3-, 5-year survival curves by the nomogram. PAAD = pancreatic adenocarcinoma.

To provide a better quantitative method for clinicians to predict cancer prognosis, a nomogram was constructed by combining the risk score with other clinicopathological risk factors. We combined the risk model with other clinical factors and performed univariate and multivariate analyses to examine the clinical independence of the model. The results showed that the risk model was able to serve as an independent prognostic indicator (*P* < .001, Fig. 6A). The nomogram showed that our risk score was an important factor among the various clinical parameters (Fig. 6B). The specific points of each parameter was shown in Table 2. The 45° line represented the best prediction. Calibration plots uncovered that the nomogram performed well (Fig. 6C). Calibration curves revealed that the predicted and actual survival rates were well matched. The total points of each patient were calculated and the patients were then divided into three subgroups including low-score, moderate-score, and high-score groups. As shown in Fig. 6D, patients in the high-score group had the worst survival rate. The predictive abilities of the nomogram were analyzed by the AUC values (AUC of 1/3/5-year OS = 0.798/0.812/0.844, Fig. 6E–G). In addition, These findings suggested that the nomogram has high accuracy in predicting overall survival.

**Table 2:** Corresponding risk score for each variable and total score.

Variables	Category	Score
Grade	G1	0
	G2	15
	G3	26
LNM	Negative	0
	Positive	75
Tumor status	Tumor free	0
	With tumor	85
Risk signature	Low	0
	High	77.5
Total score	Low risk	0-101
	Moderate risk	103.5–178.5
	High risk	≥186

### 3.6. Landscape of genome-wide mutation files in different risk models

**Figure 7.**
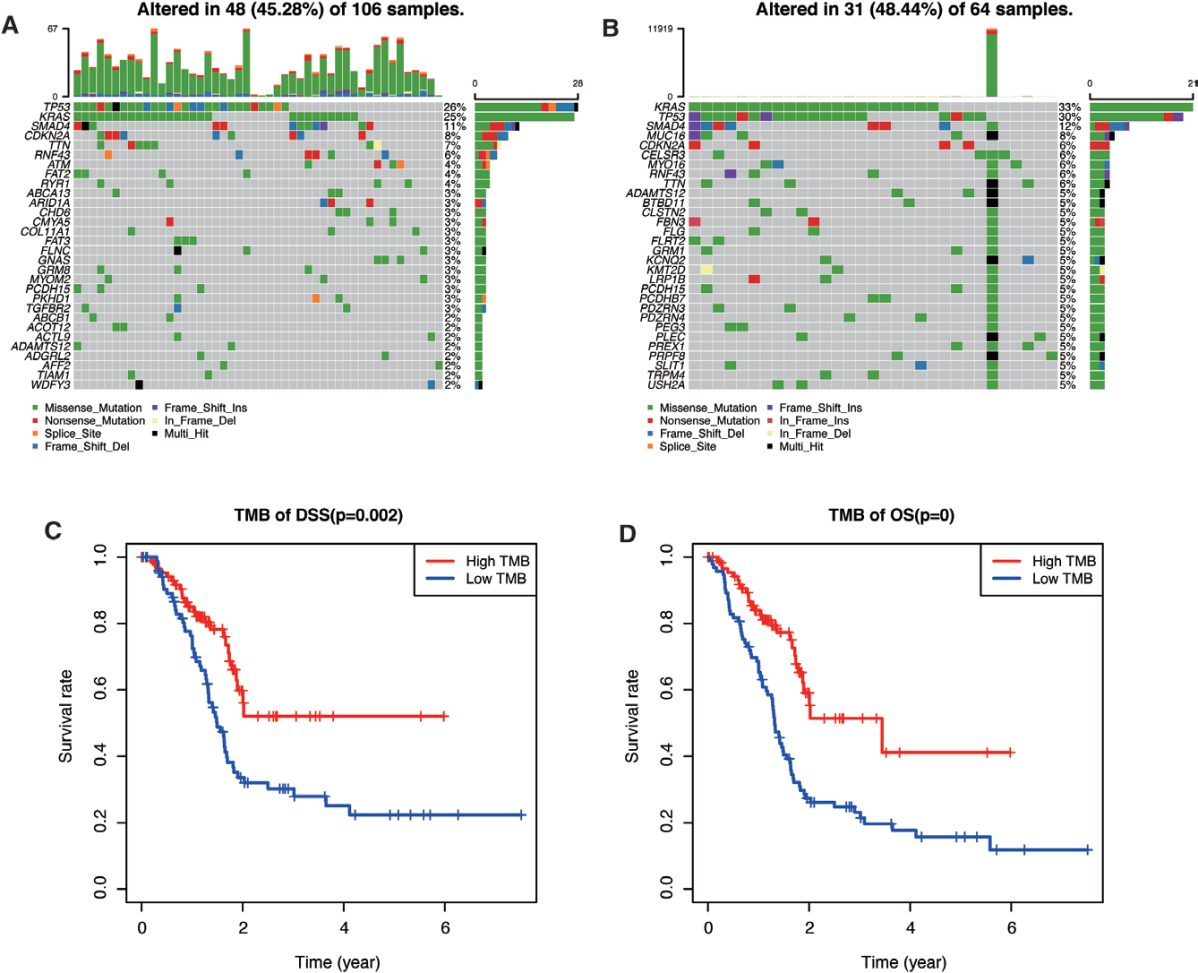
Tumor mutation characteristics in PAAD patients. Waterfall plot showing mutation profiles of patients in (A) low-risk group and (B) high-risk group. The survival analysis of patients’ (C) disease-specific survival and (D) overall survival based on tumor mutation burden scores. PAAD = pancreatic adenocarcinoma.

We obtained somatic mutation profiles of 179 patients with PAAD from the TCGA database. Mutation information of each gene in the low-risk group was shown in the waterfall plot. As for the top 30 mutated genes shown in Figure 7A, we discovered that gene TP53 mutated most frequently approximately accounting for 26%, followed by KRAS (25%), SMAD4 (11%), CDKN2A (8%), and TTN (7%). In the high-risk group, KARS mutation occupied the most frequency accounting for 39%, followed by TP53 (30%), SMAD4 (12%), MUC16 (8%), and CDKN2A (6%) (Fig. 7B). We then calculated the TMB of each patient and divided total patients into the low-TMB group and the high-TMB group by the median TMB value, and we discovered that the low-TMB group possessed worse OS (*P* < .05) and disease-specific survival (DSS, *P* < .01) than the high-TMB group (Fig. 7C,D). These results suggested that mutation characteristics in the two risk groups were different and the TMB score had a high accuracy in distinguishing the patients with worse prognoses.

## 4. Discussion

PAAD is a highly malignant tumor with a rather poor prognosis, and the 5-year survival rate for PAAD is about 5%.^[1]^ Accurate prognosis prediction can determine whether the patients benefit from more adjuvant treatment, such as intensive surgery, chemotherapy, radiation therapy, neoadjuvant therapy, and targeted molecular therapy. Traditional clinicopathological parameters have been used to reflect the occurrence, development, and important role of PAAD, and become a new therapeutic target. However, individualized treatment is urgent to improve the prognosis of PAAD patients due to their poor survival rate.

Due to the lack of effective and reliable prognostic biomarkers or models, improving the clinical prognosis of PAAD patients is still a major clinical problem. In this study, we constructed a prognostic risk model for PAAD patients based on the sequencing results in TCGA and CRGs. Seven CRGs, including RBM14, SMAD3, CENPA, KIF23, NUSAP1, INCENP, and SMC4, were used to determine the risk score of PAAD patients. It was found that the overall survival rate of patients in the high-risk group was low. By comparing the AUC of the overall survival rate, the prediction accuracy of the risk model constructed by the seven genes is very high. As some of the bioinformatics studies in PAAD have been carried out from different angles, genetic analysis has been widely used in the prediction of various types of cancer. In the past few decades, many studies have explored the prognostic model of PAAD patients. One study identified three methylated genes (SULT1E1, IGF2BP3, and MAP4K4) to construct a prognostic model. The results showed that the risk model exhibited significant prognostic accuracy for PAAD patients (AUC of 5-year OS=0.69), especially for those with advanced stage and metastatic lymph nodes.^[13]^ In another study, Wu et al established a nine-gene signature to predict the overall survival of PAAD patients. The AUC for the 3-year survival prediction of the risk model was 0.621 in the training cohorts.^[14]^ The previously mentioned 4-gene model based on transcriptome imbalance proved that the AUC of 1-year and 3-year survival in the validation group reached 0.747 and 0.695, respectively.^[5]^ However, our 7-gene model predicted AUCs of 1-, 3-, and 5-year survival rates of 0.719, 0.735, and 0.749, respectively. Therefore, compared with the nine-gene model, the prognostic features in our study obtain higher accuracy with less complexity. To the best of our knowledge, this is the first study of cell cycle-related genes (CRGs) in pancreatic cancer. The AUC of our study is higher than that of most existing studies.

In addition, we also combined the 7-gene risk signature with clinicopathological characteristics to construct a nomogram. According to the results of the time-dependent ROC curve, our nomogram showed high stability and accuracy in predicting prognosis. Our nomogram indicated that the clinical outcome prediction of 1-, 3- and 5-year OS in PAAD patients had high accuracy. These results showed that our risk model and comprehensive nomogram have high accuracy in predicting the overall survival rate of PAAD patients.

Among the seven genes identified, some of the genes have been reported to play roles in the initial and progression of various cancers. Six genes were up-regulated in the tumor group and high-risk group consisting of SMAD3, CENPA, KIF23, NUSAP1, INCENP, and SMC4. Therefore, we speculate that these genes play a role of oncogenes in the development and progress of PAAD. For example, linc00462 promotes PAAD proliferation, cell migration, invasion, and tumor metastasis through the Smad2/3 pathway.^[15]^ What’s more, KIF23, NUSAP1, and SMC4 enhanced cell proliferation and invasion and acted as potential biomarkers for the diagnosis and prognosis of PAAD.^[16–18]^ Our results showed that due to the high expression of CENPA in PAAD, CENPA was initially identified as an oncogene and confirmed that its expression was related to tumor invasiveness. CENPA has long been considered to be up-regulated in a variety of malignant tumors, especially prostate cancer, and can promote tumor growth, drug resistance, and metastasis.^[19,20]^ In our risk model, RBM14 is the only highly expressed gene in the low-risk group. RBM14 is an RNA binding protein (RBP), which can encode ribonucleoprotein and act as a nuclear coactivator and RNA splicing regulator. RBM14 prevents the assembly of the centromeric protein complex and maintains the integrity of the mitotic spindle. RBM14 also played a role as a new centromeric protein complex assembly inhibitor.^[21]^ Finally, we integrated genomic and clinicopathological features to diagnose and predict the overall survival of PAAD. In future research, we may design a rapid detection kit to indicate the expression of seven genes and calculate the risk score of each patient, to make this process simple and convenient.

Combined with GO enrichment analysis and KEGG enrichment analysis, the results suggest that these genes are closely related to the cell cycle. Cell-cycle-related genes had been reported in many other cancers such as endometrial cancer and gastric cancer^[6,7]^ Cell cycle progression increased with sequential inactivation of PAAD suppressors, yet remained higher in metastases and driven by cell cycle regulatory genes.^[22]^ Cell cycle inhibitors had outperformed in many types of tumors, and this treatment would act as a new therapeutic direction for advanced PAAD.^[23]^ In GSEA analysis, metabolism-related functions and certain pathways were found to be significant. PAAD cells have extensively reprogrammed metabolism, which is driven by oncogene-mediated cell-autonomous pathways, the unique physiology of the tumor microenvironment, and interactions with non-cancer cells.^[24]^ Hu et al found that UHRF1 promotes aerobic glycolysis and proliferation in PAAD.^[25]^ Tumor cells of PAAD utilized “metabolic reprogramming” to satisfy their energy demand and support malignant behaviors including metastasis. What’s more, PAAD cells show extensive enhancement of glycolysis.^[26]^ To better understand the specific mutant characteristics of patients in different risk groups, we conducted the mutation analysis and calculated the TMB scores. In our study, we also found that mutations of KRAS and TP53 were very common in both high- and low-risk groups. Most PAADs arise from microscopic non-invasive epithelial proliferation within the pancreatic ducts. There are four major driver genes for PAAD: KRAS, CDKN2A, TP53, and SMAD4. KRAS mutation and alterations in CDKN2A are early events in pancreatic tumorigenesis.^[27]^ What’s more, tumor mutation burden was also a significant factor for both DSS and OS in PAAD.^[28]^

Our research still has some limitations and deficiencies. First of all, since the model was developed based on sequenced expression profiles, we need more research to confirm whether the expression level of these genes is consistent with the transcription level in PAAD. Then, due to the limited clinical information of patients, we cannot conduct subgroup analysis by layering more factors. Last but not least, we still need a large, multicenter, and prospective clinical cohort to confirm the accuracy of our study, because the construction and evaluation of the prediction model are based on public data sets.

## 5. Conclusion

In conclusion, we constructed a cell cycle-related prognostic model for PAAD that can accurately predict prognosis and facilitate therapeutic decision-making and clinical monitoring. Further construction of the nomogram presented greater advantages in stability and accuracy for prognosis prediction and was promising to be applied for clinical prognostic evaluation of PAAD patients.

## Author contributions

**Conceptualization:** Dapeng Xu.

**Data curation:** Jun Shen.

**Formal analysis:** Rong Qin.

**Methodology:** Yongmin Mao.

**Project administration:** Kai Tang.

**Visualization:** Aiguo Zhang, Dafeng Wang.

**Writing – original draft:** Dapeng Xu.

**Writing – review & editing:** Yingzuo Shi.

## Supplementary Material
