# Supplementary material for: Identification of a novel cell cycle-related risk signature predicting prognosis in patients with pancreatic adenocarcinoma
Source: Medicine (Baltimore). 2022 Nov 18;101(46):e29683. doi: 10.1097/MD.0000000000029683 (PMC9678543; doi:10.1097/MD.0000000000029683)

Figure S1

Gene expression of the seven genes in different grades. (A) CENPA. (B) INCENP. (C) KIF23. (D) NUSAP1. (E) RBM14. (F) SMAD3. (G) SMC4.

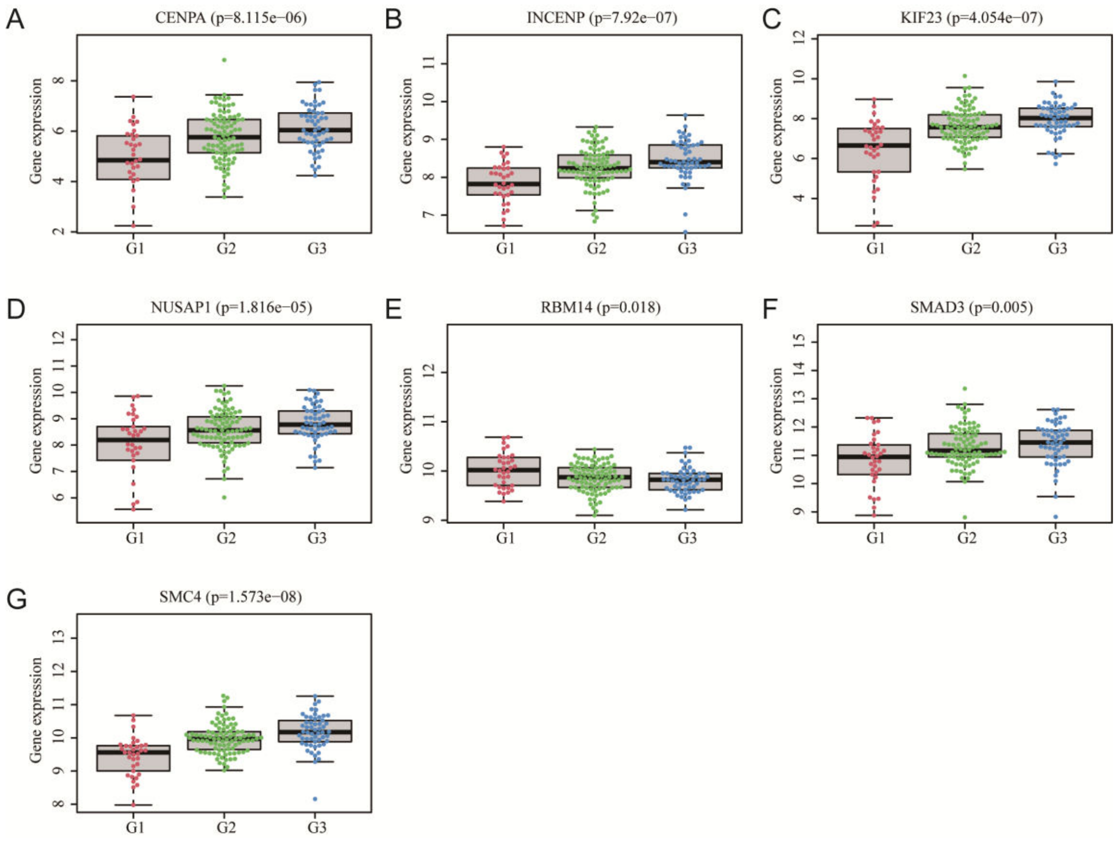

Supplement: Supplementary file 1 [file medi-101-e29683-s001.pdf]
